# Intraoperative integration of nTMS, CCEPs and DCS for language. A glance to the next future?

**DOI:** 10.1007/s00701-025-06691-5

**Published:** 2025-10-03

**Authors:** Camilla Bonaudo, Riccardo Carrai, Edoardo Pieropan, Francesca Fedi, Eleonora Visocchi, Fabrizio Baldanzi, Francesca Battista, Antonello Grippo, Alessandro Della Puppa

**Affiliations:** 1 Univeristy of Florence, Florence, Italy; 2 Neurosurgery, Department of Neuroscience, Psychology, Pharmacology and Child Health, University of Florence, University Hospital of Careggi, Florence, Italy; 3 Neurophysiopathology Unit, University Hospital of Careggi, University of Florence, Florence, Italy; 4 Department of Information Engineering, University of Padua, Padua, Italy; 5 Antares S.P.A., Dueville, Vicenza, Italy

**Keywords:** Direct-Cortical-Stimulation (DCS), Navigated-Transcranial Magnetic-Stimulation (nTMS), Cortico-Cortical-Evoked-Potentials (CCEPs), Awake surgery, Intraoperative monitoring (IOM), Low-grade-glioma surgery

## Abstract

**Background:**

Direct-Cortical-Stimulation (DCS), Navigated-Transcranial Magnetic-Stimulation (nTMS), Cortico-Cortical-Evoked-Potentials (CCEPs) can be synergically used to monitor Language-network.

**Methods:**

We illustrate a case of multimodal approach for removal of one left fronto-temporo-insular Low-Grade-Glioma.

**Results:**

A 32-years-old female patient was operated in awake surgery (using D080-picture-naming-test). We documented a matching correspondence among frontal nTMS + spots, Penfield DCS, and CCEPs stimulation strip. The same was found in the temporal area among nTMS, DCS, and CCEPs recording strip. [Average distance among nTMS, DCS and CCEPs spots = 5.10 mm, STD = 1.08 mm. Se, PPV and Precision:100%].

**Conclusions:**

We documented the potential utility of matching synergic technologies to preserve language.

**Supplementary information:**

The online version contains supplementary material available at 10.1007/s00701-025-06691-5.

## Introduction

The neurosurgical oncological domain is based on the ‘metanetwork’, composed of integrated white-matter-circuits [3]. The arcuate fasciculus (AF) is dominant for language, and its preservation is crucial [5]. Awake surgery represents the gold standard for intraoperative language testing [3], with bipolar Penfield stimulation [1]. Nevertheless, new technologies have been increasingly used to monitor the linguistic circuits, also under general anesthesia: navigated Transcranial Magnetic Stimulation (nTMS) [9, 13], and Cortico-Cortical Evoked Potentials (CCEPs) [7, 16, 20] (Table 1).

**Table 1:** Summary table of available technologies: Benefits and limitations

Technique	Invasiveness	Use	Spatial Resolution	Functional Info	Timing
DCS	Invasive	Intraoperative	High	Direct	During surgery
nTMS	Non-invasive	Pre- and post-op	Moderate	Pre- and post-op mapping	Before/after surgery
CCEPs	Invasive	Pre-op with SEEG *Intraoperative	High	Connectivity (AF connection and integrity)	Before or during surgery

*SEEG: Stereo-Electroencephalography

Integrating all these technologies can offer a more comprehensive and safer approach to brain tumor resection to optimize surgical-clinical outcome.

This work aims to share our preliminary but encouraging experience in their synergic use.

## Materials & methods

We collected data of a 32-years-old-female-patient admitted at our Neurosurgical Department. She presented nausea, dizziness and motor epileptic seizure on the right limbs. Imaging documented a left fronto-temporo-insular lesion (44x46x33mm). EEG was recorded with temporal electrodes: no gross critical activity detected. No other comorbidities reported.

The patient accepted the awake surgery. We preoperatively acquired:

- Neurocognitive evaluation: intact.
- Preoperative nTMS-motor/cognitive study was performed using Galileo NetBrain Neuronavigator 9000 system, EB Neuro Corp. Picture-naming-test was used for language [5pulses/5 Hz; intensity 100% of the first dorsal interosseus muscle (FDI) resting motor threshold (rMT)]
- MRI DTI-tractography-reconstruction was based on nTMS spots [18].

Intraoperative CCEPs were recorded using a 4-contacts-frontal strip for stimulation, while temporal lobe 4-contacts-strip was used for recording. The stimulation was carried out with 2 adjacent contacts of the stimulating strip (bipolar stimulation). The recordings were bipolar. The stimulation was carried out with biphasic-square-waves of constant-current, single-pulse electrical stimulation (pulse-weight = 0.5 ms, stimulation intensity = 20 mA, stimulation frequency = 0.9–1.1 Hz). Recordings were acquired with a hardware high-pass filter of 0.5 Hz and low-pass filter of 5 kHz. 20 ms/D sweep time was used.

Statistical analyses were performed based on the spatial coordinates (x,y,z) of DCS, nTMS and CCEPs electrodes’ spots, recorded by the neuronavigation system. We obtained a spatial distribution of nTMS and DCS points, with marginal probability densities f_XY_, f_XZ_, and f_YZ_ [2].

Ethics: our patient provided a written informed consent. The Consent to Publish Declaration was asked and signed by the patient.

## Results

**Fig. 1:**
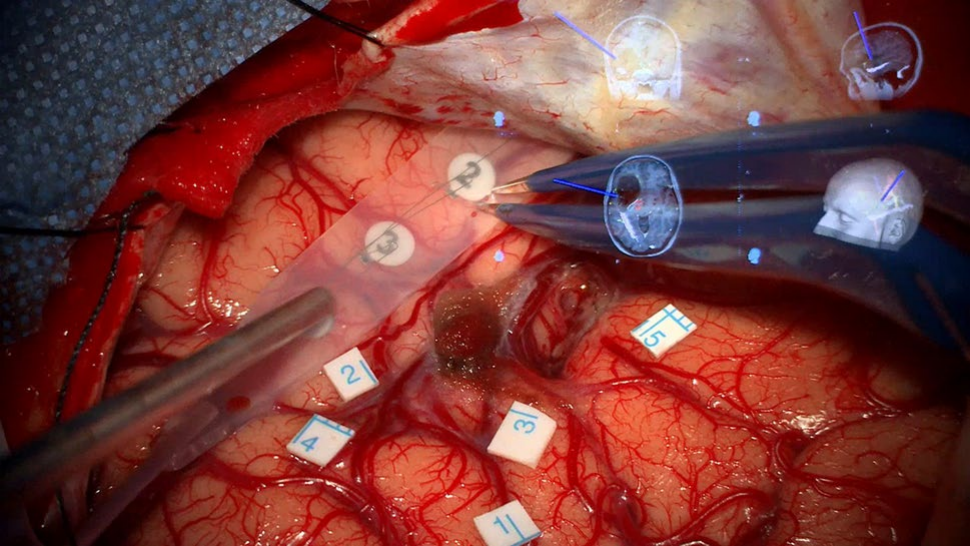
Sterile tags on the cortical surface (numbers 1 and 2) corresponding to the frontal–temporal nTMS + spots. Cortical surface and sterile tags located after DCS cortical mapping, monitoring language function: 1=speech arrest, 2=semantic paraphasia, 3=semantic paraphasia, 4=latency, 5=anomia. All errors were analysed and interpreted by our speech therapist (Dr F.F.)

Our patient was operated on in awake surgery, testing motor + language function (D080). We combined DTI-tractography, nTMS and CCEPs. Neuro-navigation figures were integrated into the dap-display screen of the microscope, always visible on the right upper corner of the screen, Fig. 1.

Surgical approach: a left front-temporal skin incision and craniotomy were performed; then, the trans-insular trans-cisternal approach was privileged. The first insular part of the tumor was removed with Cavitron ultrasound aspirator (CUSA), then temporal, and finally frontal one.

**Fig. 2:**
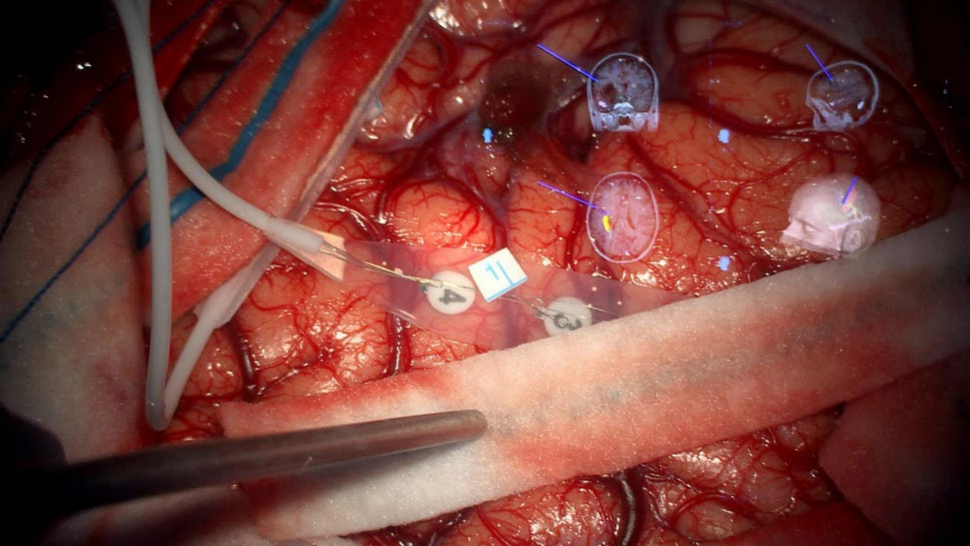
CCEPs frontal strip of stimulation: between the 3rd and the 4th electrode, we found the nTMS positive spot for semantic paraphasia and the DCS positive dot for speech arrest during Penfield bipolar stimulation

**Fig. 3:**
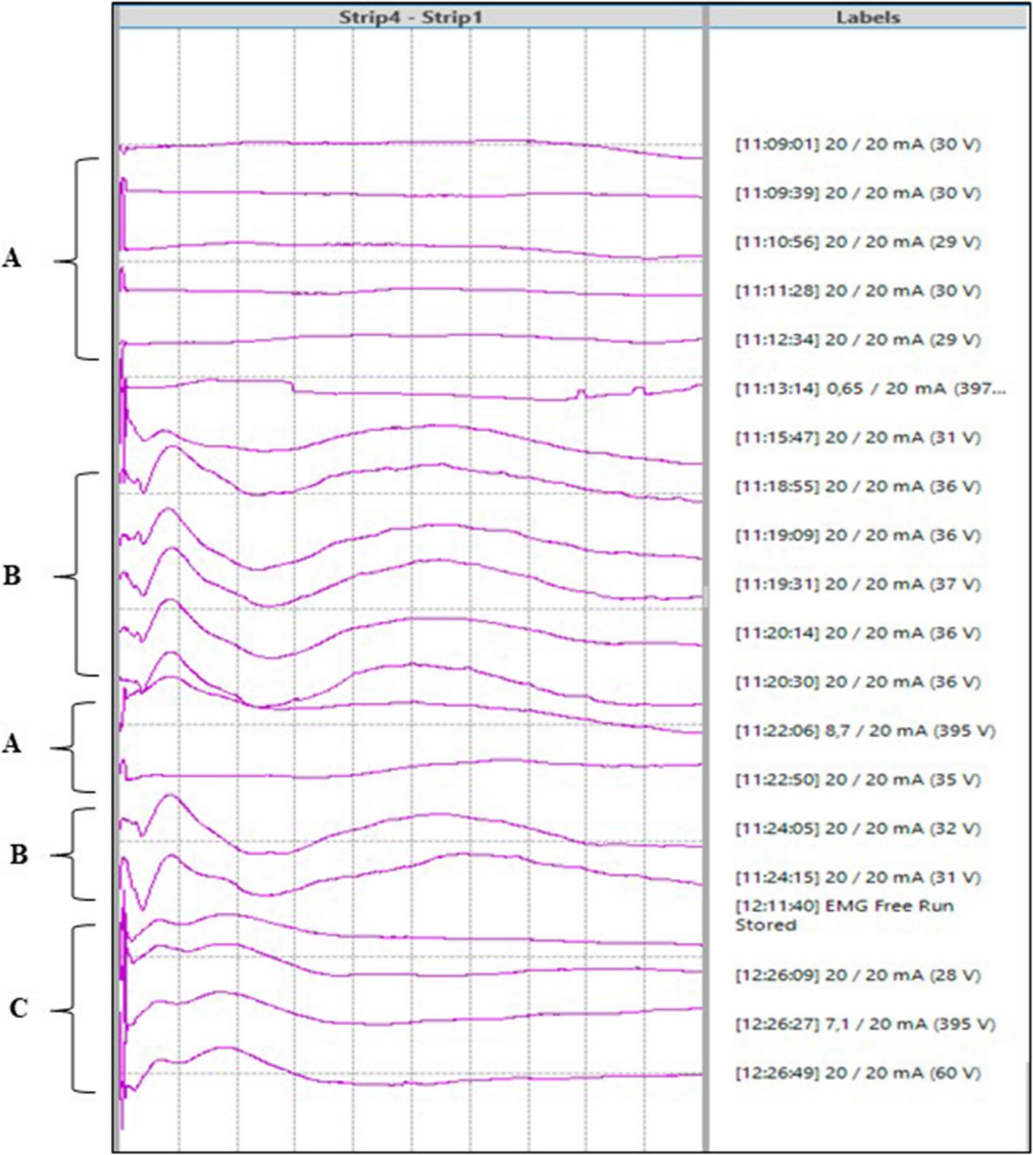
CCEPs intraoperative recording**: A** Stimulation electrodes positioned on anatomical data. **B** Stimulation electrodes placed on points determined with NTMS. **C **Stimulating electrodes placed on points determined with NTMS at the end of brain tumour removal Sens: 1200 𝛍V/D; Sweep time: 20 ms/D

We positioned sterile tags on the cortical surface (numbers 1 and 2) corresponding to the frontal–temporal nTMS + spots, anatomically located at the level of the pars opercularis of the Inferior Frontal Gyrus (IFG), and on the anterior part of the Superior Temporal Gyrus (STG), Fig. 1. At first, nTMS was used to position CCEPs strips, looking also at the branches of the AF, designed during nTMS-based-tractography-reconstruction. Then, nTMS guided DCS, so we checked the same areas with Penfield bipolar probe: we found a matching correspondence between DCS + and nTMS + spots, identifying a speech arrest during initial counting at 2 mA of electrical current intensity (tag 1, Fig. 1). The same areas were checked once more after the first round of bipolar mapping. DCS confirmed the validity of nTMS data. We documented a matching correspondence among frontal nTMS + spot for language (semantic paraphasia, tag 1), DCS Penfield stimulation spots (speech arrest + anomia, tag 1 and 5), and CCEPs stimulation strip (between the 3rd and 4th electrode). The same correspondence was found in the temporal area among nTMS spot (semantic paraphasia, tag 2), DCS (tag 4, latency), and CCEPs recording strip. CCEPs in this area corresponded to signal in trace B (Fig. 1, Fig. 2 and Fig. 3), showing a positive component (P1) at 7–8 ms, a first negative potential (N1) at 20 ms and a second negative component (N2) at 110 ms when stimulating from the frontal lobe and recording in the temporal lobe.

Figure 1 shows the final vision of the cortical surface with sterile tags (i.e. errors during language mapping) [Penfield stimulation at 2 mA].

Figure 2 reports the spatial coordinates of nTMS/DCS spots, recorded intraoperatively before and during surgery.

Therefore, we found a matching overlay between DCS, nTMS spots and the electrode that recorded a valid CCEPs (Fig. 1, 2, 3).

Afterwards, we analyzed spatial coordinates (x,y,z) of nTMS/DCS points, using the our previous statistical method [2].

**Fig. 4:**
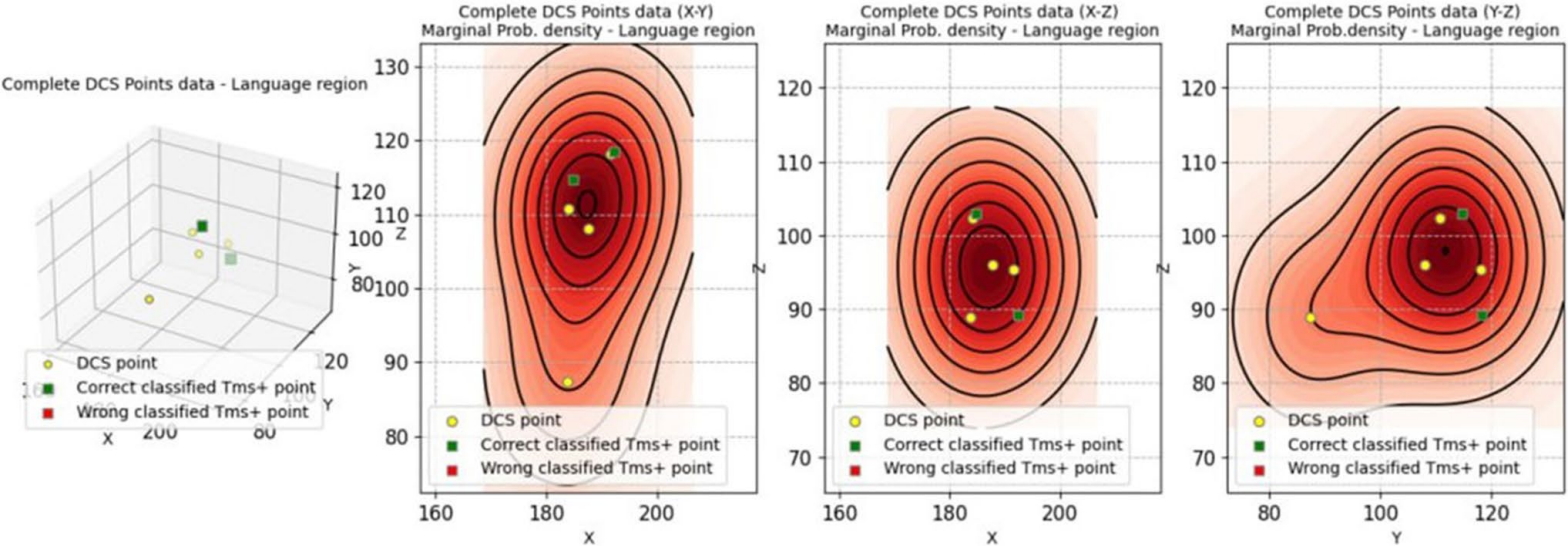
Whole spatial distributions of nTMS and DCS positive points with marginal probability densities f_XY_, f_XZ_, and f_YZ_ (95% CI; KDE, setting σ = 5 mm)

We obtained: the average distance among nTMS, DCS, and CCEPs spots was 5.10 mm, with standard deviation 1.08 mm, median 5.10 mm, interquartile range 1.08 mm. [Sensitivity, Precision and Positive Predictive Value result 100%, whereas no coordinates of negative spots were acquired, so no other statistical data can be provided] (Fig. 4 and Table 2).

**Table 2:** Spatial coordinates (x,y,z) of DCS, nTMS and CCEPs spots on the cortical surface

	Coordinates		
Tag	x	y	z
1 nTMS frontal	184.83	114.69	103.06
2 nTMS temporal	192.35	118.42	89.24
3 DCS	184.07	110.80	102.36
4 DCS	187.74	108.08	96.07
5 DCS	191.58	118.19	95.37
6 CCEPs	183.74	87.36	88.92

Sodium Fluorescein was used intraoperatively to achieve gross total resection.

Postoperative CT scan documented regular result, jeopardizing postoperative complications. The postoperative clinical outcome was unimpaired and subjectively satisfactory.

## Discussion

We present a preliminary experience first on the use of nTMS to guide either DCS and CCEPs positioning, secondly on the synergic use of multiple techniques that showed high correspondence intraoperatively: nTMS + language spots and CCEPs signal matched with the cortical area where DCS + spots were located. High precision was documented through our statistical analysis (100%).

DCS represents the gold standard during awake brain tumor surgery [4], especially for insular gliomas, to monitor several cognitive domains, with multiple simultaneous tests [15]. Nevertheless, awake procedure is not always feasible [10]. Therefore, non-invasive preoperative mapping with nTMS [19] can be used. Furthermore, CCEPs allow monitoring functional circuits under general anaesthesia [7, 20].

Concerning language function, a systematic review [9] reported that only the object-naming-task was performed in the comparative studies on nTMS and DCS, documenting Sensitivity (Se) and specificity (Sp) ranged 10–100% and 13.3–98%, respectively. The positive predictive value (PPV) and negative predictive value (NPV) ranged 17–75% and 57–100% respectively. Coherently, in a recent review [14], rTMS language mapping showed a high specificity with a very high NPV. Ille et al. [8] re-defined the concept of language eloquence, analyzing the outcomes after preoperative nrTMS-based and intraoperative DCS-based glioma resection in a cohort of 147 patients, divided into two groups (100 nTMS and 47 DCS). The functional outcome did not differ in terms of linguistic performance. Gross total resection was achieved in more cases in the nrTMS group (87%, vs. 72% in the awake group, *p* = 0.04), although the awake group obtained higher scores in the linguistic eloquence. Kram et al. [11] also proposed the comprehension-based-nTMS setup to test severe aphasic patients who would be otherwise precluded from classic production-based-mapping. Moreover, tailoring task complexity to individual performance capabilities, considering serious impairment of language, may considerably support the preservation of residual functionality in aphasic patients [12].

Whereas nTMS is repeatable pre-/postoperatively, useful also for motor rehabilitation, CCEPs have been examined to monitor AF during under general anaesthesia [6]. Giampiccolo et al. [6] operated on 9 patients for lesions in the left peri-sylvian cortex, for whom awake surgery was not indicated: CCEPs were successfully evoked in 5/9 patients. Therefore, CCEPs have promising implications in reducing deficits in patients who require surgery in language areas in asleep surgery. Specific characteristics of CCEPs have been analyzed also by Seidel et al [17], documenting significantly different latencies among glioma grades and demonstrating that higher latency in IDH-wild-type-Glioblastomas may represent a more infiltrative disease. Some aspects of CCEPs are still debated, since on one hand the monitoring of cortico-cortical connections opens a new pathway for tract monitoring, beyond AF [7]; on the other hand, it remains unclear whether they are a reliable method of continuous intraoperative monitoring of speech function, because of lack of post-operative outcome. Many works record CCEPs first and then re-analyze them after surgery, not using them as an early indicator of clinical impairment.

As far as we know, no studies about a comparison between DCS, nTMS and CCEPs have been published so far. Although the limitations of the present technical case (i.e. single case, no statistical significance), we would like to highlight the utility to apply multiple technologies to implement quality of surgery, minimizing the risk of postoperative clinical impairment and maximizing safe resection. Moreover, we emphasize CCEPs were done before Penfield DCS, which was an a-posteriori proof of the validity of nTMS data. Based on the postoperative neurological integrity of our patient, we can conclude that CCEPs’ slight modification between the phase before and after excision was related to a favorable postoperative outcome.

Our data supports the integration of different technologies, stressing the potential utility of nTMS guidance for DCS and CCEPs recording to study in real-time language circuits, jeopardizing injuries and optimizing clinical outcome.

## Conclusions

This preliminary work documents the utility of multiple intraoperative monitoring, showing correspondence between CCEPs and nTMS for language, compared to the gold standard DCS. Further data is needed to validate our preliminary but promising results.

## Supplementary information

Below is the link to the electronic supplementary material.

ESM 1 Supplementary Material 1 (JPG 223 KB)

ESM 2 Supplementary Material 2 (JPG 173 KB)

ESM 3 Supplementary Material 3 (JPG 109 KB)

ESM 4 Supplementary Material 4 (JPG 129 KB)

ESM 5 Supplementary Material 5 (DOCX 15.9 KB)

## Data Availability

No datasets were generated or analysed during the current study.
