# Supplementary figures and images for: Intraoperative integration of nTMS, CCEPs and DCS for language. A glance to the next future?
Source: Acta Neurochir (Wien). 2025 Oct 3;167(1):263. doi: 10.1007/s00701-025-06691-5 (PMC12491366; doi:10.1007/s00701-025-06691-5)

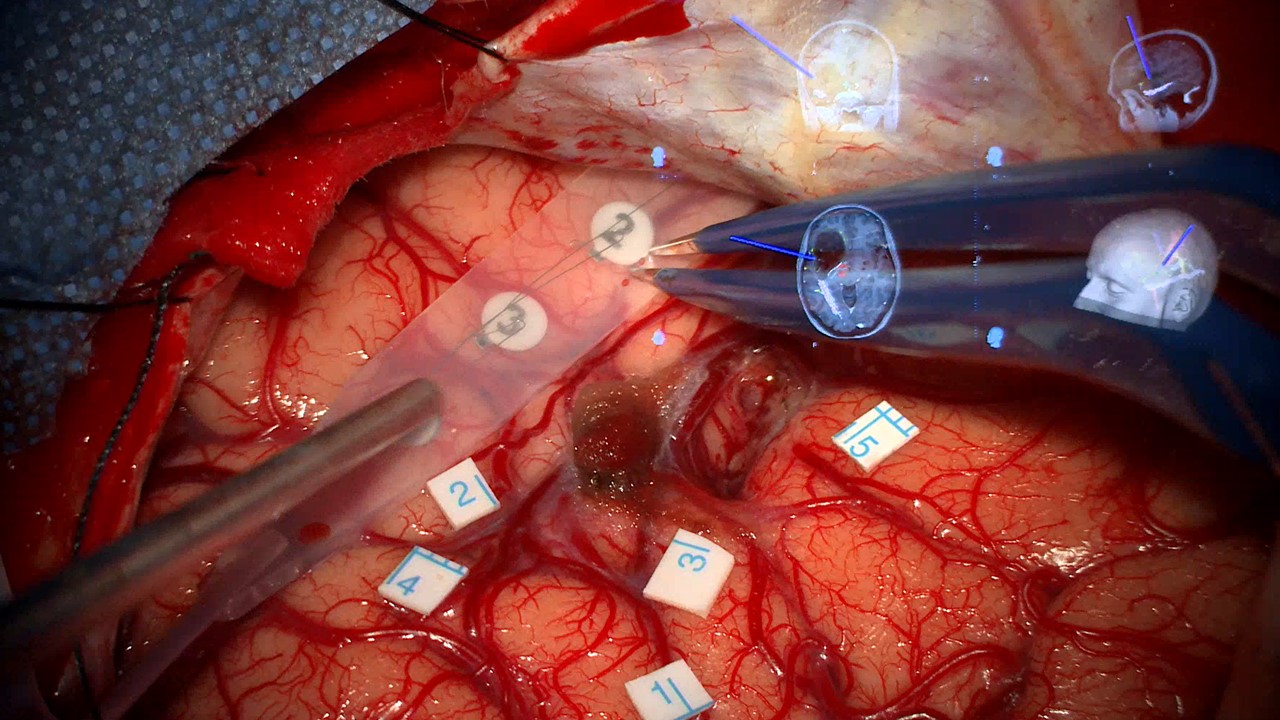

Supplement: Supplementary file 1 — Supplementary Material 1 (JPG 223 KB) [file 701_2025_6691_MOESM1_ESM.jpg]

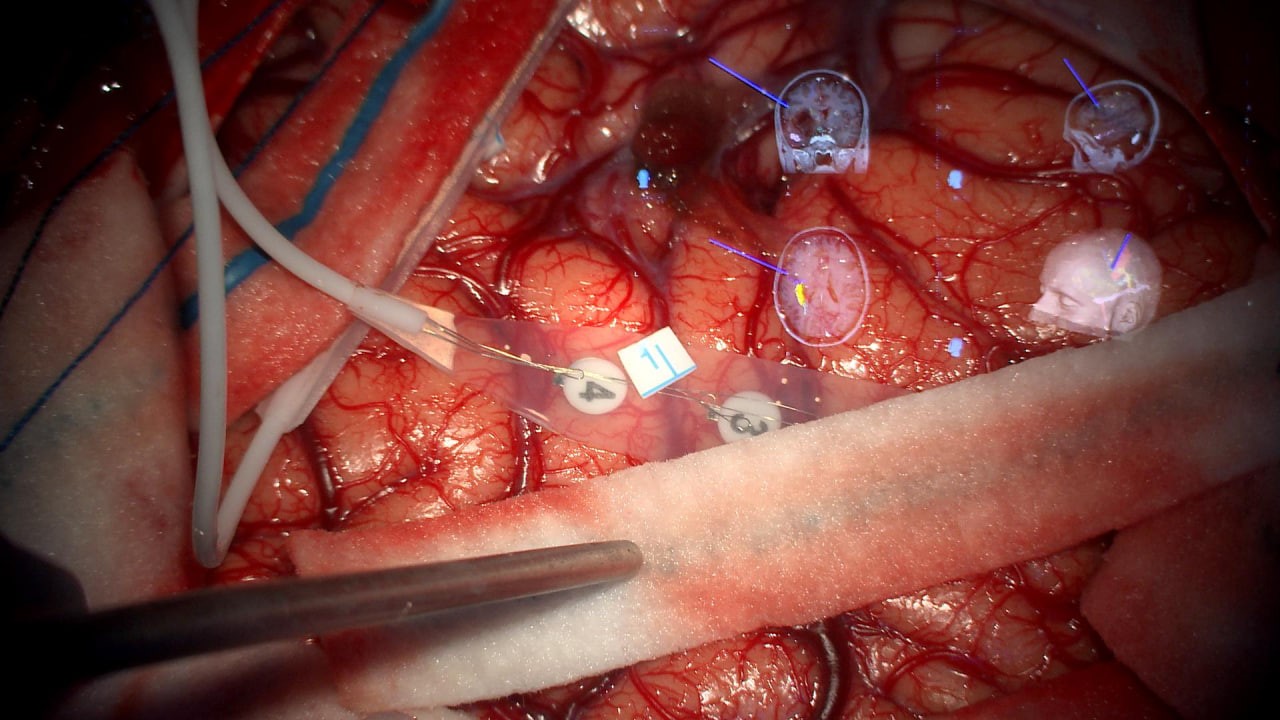

Supplement: Supplementary file 2 — Supplementary Material 2 (JPG 173 KB) [file 701_2025_6691_MOESM2_ESM.jpg]

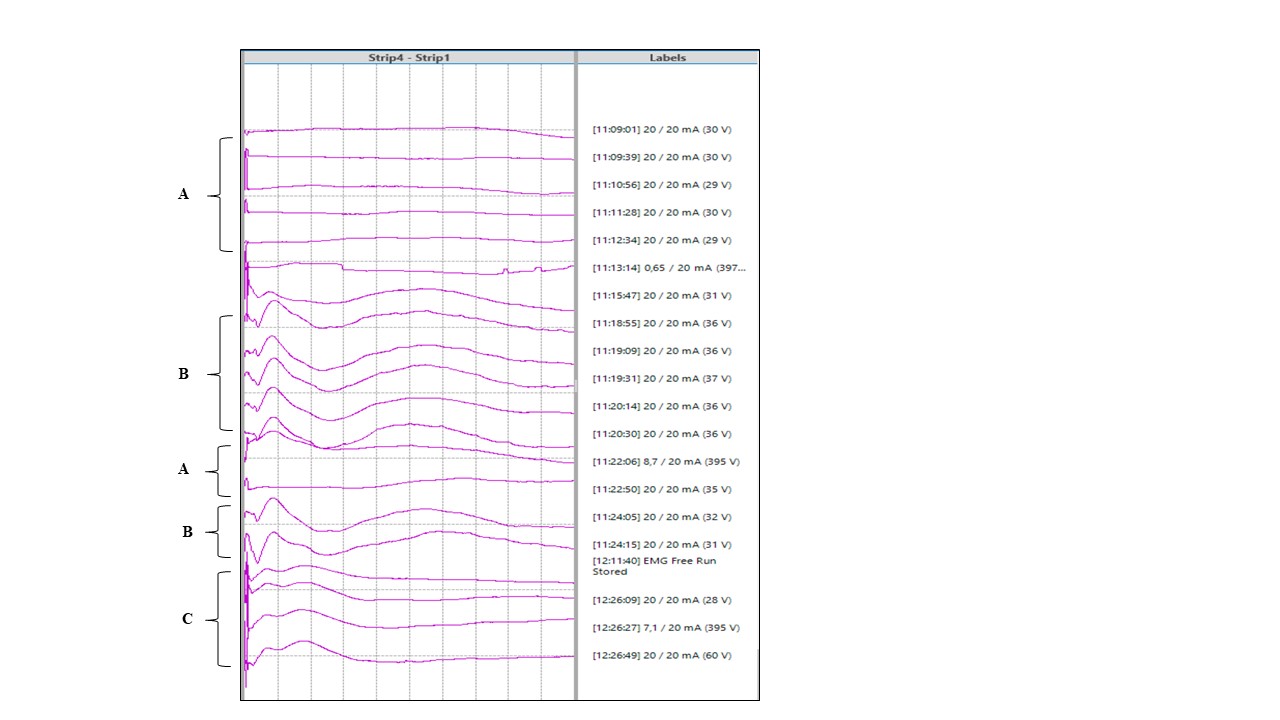

Supplement: Supplementary file 3 — Supplementary Material 3 (JPG 109 KB) [file 701_2025_6691_MOESM3_ESM.jpg]

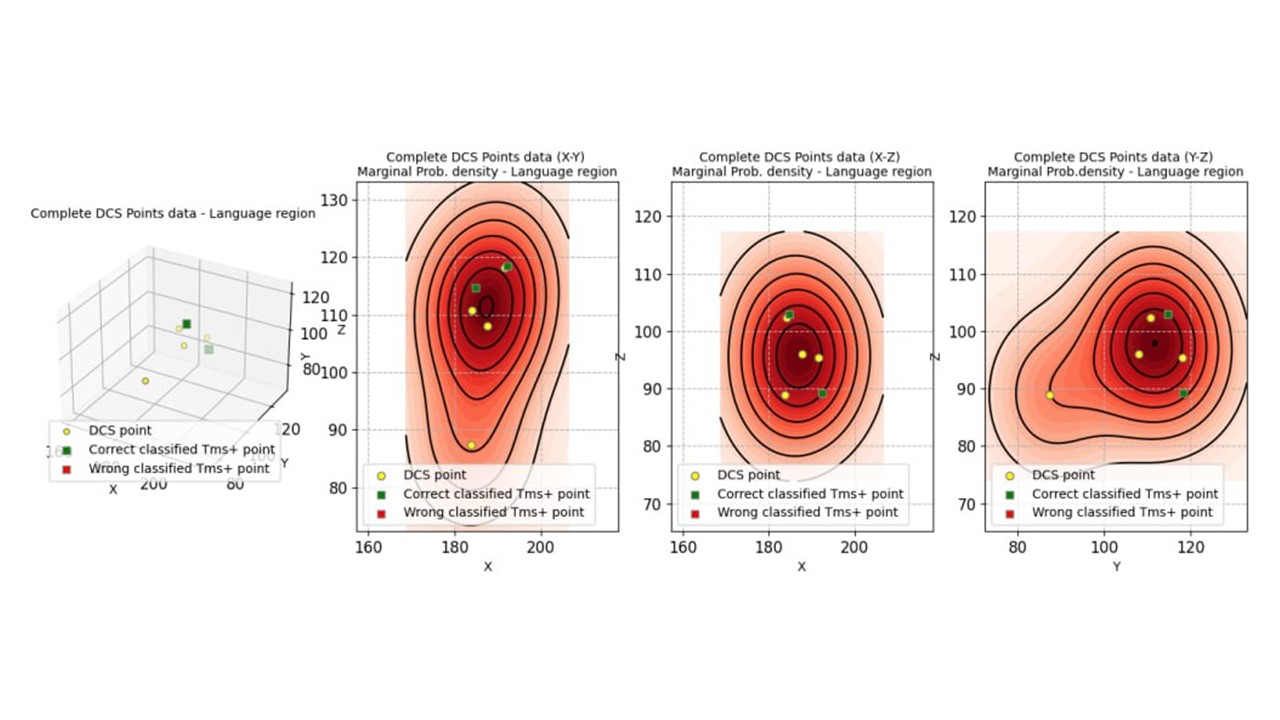

Supplement: Supplementary file 4 — Supplementary Material 4 (JPG 129 KB) [file 701_2025_6691_MOESM4_ESM.jpg]
