# Supplementary material for: Intraoperative integration of nTMS, CCEPs and DCS for language. A glance to the next future?
Source: Acta Neurochir (Wien). 2025 Oct 3;167(1):263. doi: 10.1007/s00701-025-06691-5 (PMC12491366; doi:10.1007/s00701-025-06691-5)
Supplement: Supplementary file 5 — Supplementary Material 5 (DOCX 15.9 KB) [file 701_2025_6691_MOESM5_ESM.docx]

**TABLES**

**Table 1:** Summary Table of available technologies: Benefits and Limitations

| **Technique** | **Invasiveness** | **Use** | **Spatial Resolution** | **Functional Info** | **Timing** |
| --- | --- | --- | --- | --- | --- |
| **DCS** | Invasive | Intraoperative | High | Direct | During surgery |
| **nTMS** | Non-invasive | Pre- and post-op | Moderate | Pre- and post-op mapping | Before/after surgery |
| **CCEPs** | Invasive | *Pre-op with SEEG**  Intraoperative | High | Connectivity  (AF connection  and integrity) | Before or during surgery |

**SEEG: Stereo-Electroencephalography*

**Table 2**: Spatial coordinates (x,y,z) of DCS, nTMS and CCEPs spots on the cortical surface

|  | Coordinates |  |  |
| --- | --- | --- | --- |
| Tag | x | y | z |
| 1 nTMS frontal | 184.83 | 114.69 | 103.06 |
| 2 nTMS temporal | 192.35 | 118.42 | 89.24 |
| 3 DCS | 184.07 | 110.80 | 102.36 |
| 4 DCS | 187.74 | 108.08 | 96.07 |
| 5 DCS | 191.58 | 118.19 | 95.37 |
| 6 CCEPs | 183.74 | 87.36 | 88.92 |
